# How decontextualised risk information affects clinicians’ understanding of risk and uncertainty in primary care diagnosis: a qualitative study of clinical vignettes

**DOI:** 10.3399/BJGPO.2025.0040

**Published:** 2025-12-19

**Authors:** Alex Burns, Elizabeth Shephard, Raff Calitri, Adrian Mercer, Edmund Jack, Mark Tarrant, Sarah Dean

**Affiliations:** 1 Medical School, University of Exeter, Exeter, UK; 2 University of Plymouth, School of Psychology, Plymouth, UK; 3 Patient and Public Contributor, Devon, UK; 4 Peninsula Medical School, Plymouth, UK

**Keywords:** primary health care, diagnosis, uncertainty, general practitioners

## Abstract

**Background:**

Decontextualised risk information (DRI) is any information pertaining to diagnosis, which is introduced into a clinical consultation, or a diagnostic thought process, without being requested by the clinician. It can be risk scores, computerised warnings, or laboratory tests or diagnostic imaging requests ordered by other clinicians. It is an increasing, and yet under-researched phenomena in UK primary care.

**Aim:**

To investigate how GPs integrate DRI into their clinical decision making and how might they communicate this to patients.

**Design & setting:**

Clinical vignettes of cases that involve DRI, designed to increase the diagnostic uncertainty of the case, were presented to UK trained GPs. 'Think-aloud' techniques and qualitative interviews were used to explore clinical thinking.

**Method:**

Nine GPs were interviewed. After a warmup vignette, clinicians were shown and asked to talk through three clinical vignettes, which involved DRI. Semi-structured interview questions, exploring diagnostic thinking and uncertainty, followed each vignette. Thematic analysis was used to explore the research question.

**Results:**

DRI tends to dominate a consultation when introduced. It can produce cognitive dissonance, defensive medicine, and more complex consultations. DRI explicitly presents differential diagnoses that clinicians may have considered but not discussed, compelling them to act, or justify their inaction, at several levels. Clinicians needed to recognise the complexity of clinical reasoning, and balance this against over-reliance on individual test or risk scores.

**Conclusion:**

When DRI conflicts with a clinician’s judgement, it can produce cognitive dissonance leading to complex consultations and predisposes towards defensive medical practices.

## How this fits in

Decontextualised risk information (DRI) is a new conceptualisation of a situation that will be familiar to many clinicians: the introduction of diagnostic data without a clinician requesting it. This is, to the authors’ knowledge, the first time this has been described. While DRI is intended to reduce the risk of missed diagnoses, when such information conflicts with clinical judgement, it can complicate consultations. It can make them more complex, less patient-centred, and predispose clinicians towards defensive medical practice.

## Introduction

*Mrs B, with a history of anaemia from heavy menorrhagia, presents with progressive shortness of breath and renewed heavy bleeding. Blood tests confirm significant anaemia, but a previously ordered D-dimer test for pulmonary embolism is positive. You have not ordered this blood test, and do not think it was needed, but now need to process it …*

The standard medical consultation involves a patient describing symptoms to a clinician who then asks questions and may perform a physical examination.^1,2^ At this point, a decision is made on whether additional information is needed to clarify the diagnosis. Such information could be diagnostic tests or clinical risk scores,^3^ which combine data, such as observations, demographics, and laboratory results, to assist in diagnosis.

A patient’s symptoms, personal experiences, and preferences fall within their own epistemic domain. It is an area of knowledge and understanding that rightly belongs to them: the reason they seek medical attention.^4,5^ In contrast, decisions about further testing require specialist medical knowledge and are generally recognised as ultimately belonging to the clinician’s epistemic domain.^5,6^ A clinician's decision to seek further risk information is critical. For some diseases such as cancer, the decision to test is often more predictive than the test outcome itself,^7–9^ highlighting the importance and predictive power of clinician gestalt.

The predictive value of any diagnostic risk information depends on the discriminatory characteristics of the risk information (it has to be good at distinguishing between disease and no disease), but also on the pretest probability.^7–9^ Pretest probability is an estimation, combining research evidence and clinical judgement.^10^ It is refined by taking account of the patient’s history and examination, both of which are subjective inputs influenced by the clinician’s interpretation and experience. Any pretest probability is a broad, tacit estimate, and is likely to vary among clinicians. The decision on whether and when to discuss ordering further tests with a patient is influenced by this pretest probability, its associated uncertainty, and the clinician’s risk tolerance. The process is dynamic, and vulnerable to cognitive biases^11^ such as availability bias,^12^ where a diagnosis is deemed likely owing to recent clinical experience of it, and anchoring bias, ^13^ where a clinician over-relies on the initial information they receive. It can result in 'defensive medicine', a departure from sound medical practice in which tests or treatments are ordered not for the patient’s benefit but to shield the clinician from potential complaints or litigation.^14^ Unsolicited DRI has the potential to challenge the clinician’s clinical decision making, influencing the uncertainty present in any consultation.

We define DRI as risk information that is presented out of sync with when a clinician would normally gather such information, and out of the context of their diagnostic thought processes. Patient-driven DRI, such as private testing^15^ and data from wearable technology,^16^ is rising in frequency, consuming considerable GP time.^17^ UK policy seems to promote an increase in future use.^18^ System-driven DRI, such as automated clinical scores (for example, National Early Warning Score 2 [NEWS2])^19^ and computer-triggered electronic risk scores,^20,21^ is also increasing. Progressive decline in continuity of care^22^ makes it less likely for the same clinician to request and process the same test. While DRI aligning with a clinician’s diagnosis poses little challenge, conflicting DRI may create uncertainty.

In primary care, where disease prevalence is lower than other settings,^23,24^ diagnostic tests and DRI are less predictive and more prone to false positives. This makes forgoing additional testing or risk scoring more reasonable in this setting. This merits its investigation in a primary care setting.

### Aim

The aim of this study is to explore how DRI affects clinical reasoning in the primary care consultation when it conflicts with the initial clinical hypothesis, and how might it affect communication with the patient.

### How patients were involved in this research

The study concept was discussed with a patient and public involvement and engagement (PPIE) group arranged by PenARC (Applied Research Collaboration, South West Peninsula). The interview guide and emerging themes were discussed with and reviewed by AM before finalisation, who also contributed to writing this report.

## Method

Three clinical vignettes^25^ (including Mrs B above) were created to simulate DRI, based on AB’s GP experience and reviewed by EJ and four other GPs. Details were adjusted to reflect typical UK primary care cases. Each scenario featured a potentially serious diagnosis (sepsis, pulmonary embolism, or lung cancer), but in a low-risk 'context', designed to suggest a conservative management plan without immediate hospital admission or urgent referral. DRI was then introduced using a NEWS2 score (sepsis),^26^ a D-dimer test (pulmonary embolism), and a cancer risk assessment tool^27^ (lung cancer). These tests represent 'decontextualised' information: not sought by clinicians in low-risk cases. During pilot testing, when delaying a decision was an option, clinicians often chose to wait or repeat tests. The potential diagnoses were therefore chosen to be serious, time-urgent conditions requiring clinicians to decide either to immediately act on the DRI or disregard it.

Recruitment was through adverts to the Cornish and Devon Local Medical Committee (organisations that represent GPs within a geographical area), memberships via a monthly email (Cornwall), and a website post (Devon). Responders received consent forms and study details by email. AB interviewed each GP via Microsoft (MS) Teams, recording verbal consent beforehand. Cameras were optional (one GP turned theirs off). AB presented vignettes using PowerPoint slides (see Appendix). A warmup vignette without DRI introduced the 'thinking-aloud' method,^28,29^ which captures clinicians' real-time reasoning by having them vocalise their thoughts during a task. This technique potentially offers insights by recording clinical decision-making processes in real time.

The clinician reviewed each study vignette, explaining their diagnostic reasoning and management plan, including tests, treatment, or hospital admission. DRI was then introduced, prompting discussion on its impact on their reasoning, adjustments to the plan, and communication with the patient. After the three cases, further questions explored real-world experiences with DRI.

MS Teams' automatic transcripts were reviewed and corrected by AB. Transcripts were read, re-read, and inductively coded by AB using NVivo (version 14) software.^30^ An independent coding was performed by SD with three of the transcripts. Analysis followed through thematic analysis^31^ with a reflexive approach, acknowledging AB’s role as a GP and prior acquaintance of some participants.^32^ A critical realism perspective^33^ was adopted, assuming that serious diagnoses exist independent of human observation. During a clinical consultation, symptoms, signs, tests, and — as in this study — DRI, act as perceptual layers, providing an incomplete and imperfect view of that underlying reality.

The coding process began with broad initial codes capturing aspects of diagnostic decision making, risk, uncertainty, and communication with patients. Related codes were grouped into emerging themes, which the research team reviewed to identify overlaps or deeper meanings reflected in language or metaphors. The concept of 'information power'^34^ guided the evaluation of the data’s relevance and contribution to the study’s aims, and was deemed sufficient for addressing the research question within the study’s practical and financial constraints. Themes were refined further, leading to the results presented below.

## Results

Ten GPs responded to the invitation. Nine were recruited, all from Cornwall, after one was unable to commit. No clinicians withdrew once scheduled. Interviewee characteristics are presented in Table 1. Average interview length was just under an hour.

**Table 1.** Participant characteristics

Characteristics of participant		Number of participants
Sex	Female	5
	Male	4
Professional role	GP partner	2
	Salaried or locum^a^	3
	GP ST3 trainee	4
Total years of clinical experience (since medical qualification)	>20 years	1
	15–19 years	3
	10–14 years	3
	5–9 years	2
Years of clinical experience in primary care	>15 years	1
	10–14 years	3
	5–9 years	1
	<5 years	4
**Self-reported risk and uncertainty tolerance**		
Compared with your peers, are you more or less tolerant of risk?	More risk tolerant than peers	5
	About average	0
	Less risk tolerant than peers	4
Compared with your peers, do you order fewer, or more tests?	More than peers	2
	About average	4
	Fewer than peers	3
Compared with your peers, how well do you handle uncertainty?	Better than average	4
	About average	3
	Worse than average	2
**Prior relationship to participant**	No prior relationship	5
	Had met before^b^	2
	Had worked together	2

^a^None of the participants were purely salaried. All performed locum roles in addition. ^b^Participants who AB had met on one or more occasion before, but not been work colleagues. For example, contact in educational sessions

The following terms are used for clarity: the 'initial' diagnosis refers to the benign working diagnosis formed before DRI introduction. The 'serious' diagnosis is the one suggested by DRI. To 'override' DRI means maintaining the initial diagnosis without further investigation.

Before DRI, clinicians planned to proceed without hospital admission or urgent referral, perceiving a low-risk diagnosis and not seeking DRI (except GP09, who requested a NEWS2 score in the first vignette). As intended in the vignette design, DRI conflicted with the initial diagnosis in each case.

The following themes were developed: 'DRI takes over the consultation'; 'Justification and trust'; and 'Rarely black and white ... steer through the nuance'.

### Theme 1: DRI takes over the consultation

Once DRI was introduced into the consultation, its consideration, discussion, and mitigation became the focal point of the consultation:

*'I mean, I think my heart sinks because I feel you have to obviously discuss it with the patient, and you probably have to do something about it.'* (GP09)

Clinicians faced a frustrating internal conflict between their professional judgement, which deemed DRI unnecessary, and pressures to act defensively or comply with the DRI risk indicator:

*'That’s a ridiculous thing, isn't it? It hasn't changed it* [the likely diagnosis]*, but it still changes how you feel about the consultation and how the patient might feel about the consultation then.'* (GP08)

While clinicians acknowledged that the DRI could have value in raising awareness of severe yet overlooked conditions — *'It’s not unreasonable to think about serious things*' (GP05) — they felt it challenged their professional expertise and decision making:

*'I’d made a plan that was safe and reasonable, and then the extra information* [DRI] *… makes you question your original assessment and decision … I don’t know how helpful that is because I think most of the time … we probably are quite sensible as GPs.'* (GP09)

The DRI also seemed to intensify the clinicians’ worry about the threat of legal vulnerability. It forced them to grapple with clinical decisions, which were fear and anxiety inducing:

*'GP is full of uncertainty … and you’re constantly being faced with these numbers and scoring systems* [DRI] *that make you think twice and doubt yourself, and doubt your colleagues and cause uncertainty and terror.'* (GP07)

*'The three little bits of extra information* [DRI] *just kind of highlight the fact that you're taking a risk here. You are taking a risk here and it’s not very comfortable.'* (GP09)

These reactions can be understood as one of cognitive dissonance, the psychological state that arises when an individual experiences conflict between their actions, beliefs, or principles. Clinicians described being compelled to act on the DRI, which forced uncomfortable trade-off when they felt it contradicted their clinical judgement and their principle of prioritising the patient’s best interests. This incongruence between their professional identity and the behaviours they felt forced to adopt incited discomfort:

*'We can’t ignore it … I just feel like I’m being mechanical, and I don’t want to be like that … it brings me to doing Defensive Medicine, which feels awful.'* (GP05)

Fear of harm to patients under their care was a recurring theme, compounding the cognitive dissonance clinicians experienced. They felt that fear-driven decisions were not ideal, but acknowledged its influence on their actions: *'I do think that fear probably plays much more than it should. Actually, I don’t think it’s ever particularly helpful in medicine'* (GP09). At times, this fear led to defensive practices, such as unnecessary referrals or testing, aimed at mitigating potential worst-case scenarios.

Conversely, some clinicians dealt with the DRI-generated dissonance by consciously overriding or ignoring it. Here, a clinician overrides it as part of their professional identity:

*'I’ll take the risk … we’re the ones who are supposed to be able to carry the risk and make … a sensible decision. I think that’s why our job is difficult actually'* (GP09).

Another clinician took an extreme but pragmatic approach, arguing that the constraints of their working environment rendered DRI impractical:

*'I just dismiss it* [the DRI] *… It comes at a risk, it really does, but you kind of have to ignore that'* (GP03).

### Theme 2: Justification and trust

The DRI introduced a communication challenge into the consultation. A serious diagnosis, previously considered privately by the clinician but not disclosed to the patient, now requires further discussion.

This tension manifests across several levels: communication with the patient, justification to peers, and accountability to the wider system. All decisions required justification, and also an element of trust.

#### Communication with the patient

When overriding DRI, clinicians experience a tension between their clinical reasoning and the risk of withholding information from the patient. To resolve this, some clinicians engage in shared decision making, transferring part of the responsibility to the patient:

*'… we're almost handing that decision back to the patient … allows us to share that decision with them.'* (GP07)

This strategy reduces the discomfort of unilateral decision making. In the example of Mrs B, even when a patient attempted to defer the decision back to the clinician, they stressed the importance of patient involvement:

*'Gone are the days when we just tell you what to do, Mrs. B …*' (GP08)

Clinicians must balance this communication with their trust in the patient. If they are going to override the DRI, they must be confident that a patient *'… is open and willing to engage in a conversation about this and isn't likely to sue my ass if I get it wrong. You know that that that is a factor in all of this, isn't it? … can we have a conversation that is informative and open with the patient? And if I get the slightest feeling that she has a litigious propensity or has issues either with me or the practice, then I may well … this isn't clinically right … I may well treat this case a little differently …. more defensive* ... ' (GP01).

#### Justification to peers

Clinicians placed value on the discussion of their decision with a peer. It reassured them and allowed them to trust that their decisions were reasonable:

*'… I'm then sharing the risk with another clinician … then I've kind of documented a conversation with someone else too.'* (GP08)

*'I usually … discuss it with a sensible colleague and just say “look, neither of us think this should be acted upon.”*' (GP09)

GPs also considered the backing of a colleague to be a defence against the medico-legal threat of the DRI. Below the quotes refer to the 'Bolam principle', which states that an act is not negligent if clinicians of similar standing and expertise would have acted in the same way:

*'… the way we practice is … somewhat influenced by “what’s the likelihood if I get this wrong, it’s gonna go really badly for me?”… the bar that we're all taught at medical school 20 years ago was, “would a body of your peers do something similar in this situation?”*' (GP01)

*'… if a group of doctors looked at what you'd done and felt that it was reasonable, you know, you haven't been negligent.'* (GP09)

Clinicians recognised that there was often poor empirical evidence for complex clinical decisions, and that this legal definition was a good compass for quality care:

*'… what does that legal threshold relate to? … I feel that acknowledges that we don't have an evidence base for every diagnostic risk stratification decision that we make ... There is some benefit in looking at, well, “what would most of us do with this based on all of our collective experience?"'* (GP01)

#### Justification to the wider system

A clinician must finally justify their decisions to the wider system, which determines whether negligence occurred in the event of a missed diagnosis:

*'What I'm worried about might happen is actually that* [NEWS2 score] *“five” translates to a sepsis. Now, if that “five” wasn't there, it still might translate to a sepsis, but it’s been documented. Yeah. And therefore when it gets read by whoever might be interested in it, they would have something to say about that*.' (GP02)

DRI was then seen as a manifestation that this wider system lacks trust in clinicians:

*'… we get so many pop ups on our screens that to me this is just another one of those … where the powers that be assume that we are thick and don't know what we're doing … it’s not helpful. I'm not stupid. Leave me alone.'* (GP03)

### Theme 3: 'Rarely black and white … steer through the nuance'

This theme explores how clinicians can incorporate DRI into their decision-making processes while minimising cognitive dissonance. Clinicians acknowledge that decision making is a nuanced and complex process, requiring the integration of diverse information sources. Good decision making rarely relies on a single datapoint.

Clinicians recognised that DRI can be a challenge to their clinical thinking, as it adds an apparent objective 'fact' at part of the consultation where it is not wanted:

*'… the way we are all taught to practice medicine is that you see the patient, you do the history and examination and you then request the test that you think are needed and increasingly we're moving away from that model for the sake of efficiency …. I think it’s very hard for people without experience to ignore* [the DRI] *in the way that I have … I don't think I bring any kind of cerebral specialism to this. I think I just bring a few years of experience having done this a few times, been around the bend a few times.'* (GP01)

They were concerned that, in the event of an error, criticisms of their decision making *'will attempt to hang a justification on a single digit'* (GP02).

One way to avoid this simplistic thinking was to recognise and make explicit the complexities of clinical decision making. Clinicians felt they should try to help a patient to *'step outside the binary into the grey areas with me … in that non-binary area of thinking, where you're talking nuance, you're talking weighing things up …'* (GP02)

The process of *'stepping outside the binary'* was achieved in several ways. Some clinicians rationalised away the DRI. This could be via questioning it epistemologically:

*'Well, I don't think that’s* [the NEWS2 score] *validated in primary care.'* (GP03)

Or, in the case of clinical scores, it could be by looking at the component clinical features that lead to the DRI: in effect deconstructing the score as a way of rationalising its import. Here a clinician examines the individual components of a NEWS2 score to justify their lack of concern:

*'OK. I think that none of his parameters are too far out, so I think a lot of them …. what would be helpful is to have a baseline … like he may well always have a slightly low blood pressure, he may well always have a slightly high pulse. In fact, we've got some evidence for that … they're not so far out that I think he’s in imminent danger of dying from sepsis.'* (GP09)

And here another clinician justifies the lack of action with the lung cancer case:

*'So in my head I'll go through it again. Like, look, I'm reassured. We've got reasons and for the weight loss, we've got a daily cough, but he’s got severe COPD.*' (GP06)

Clinicians had the opinion that a holistic clinical judgement was better than any single piece of risk information.

*'I think it* [DRI] *can't look at the subtle things we were talking about. And put that all together. And neither does it have that gut feeling that is actually quite accurate.'* (GP09)

Ultimately, good clinical judgement, free from fear, was viewed as both an enjoyable aspect of their work and a core part of their identity as clinicians.

*'Yes, because it’s all in the clinical nuance. That’s what I love about the job … it’s very rarely black and white. It’s all in the big bubble of grey that it’s more art than science …. And when you're talking to a doc who sees things the same, it’s a really enriching conversation you have about patients. You could just really just steer through the nuance, and you know damn well that it beats hands down any risk score. And actually, what …* [DRI] *ultimately does is lends some form of exterior sort of light bulb, for you to just think a minute what we're talking about.'* (GP02)

## Discussion

### Summary

DRI, when it conflicts with a clinician’s working diagnosis, tends to dominate consultations. It makes clinical reasoning more complex, can complicate how diagnostic thinking is communicated, and increases the likelihood of defensive medical practices. Clinicians can manage it by recognising their expertise and the nuances of medical diagnosis.

### Strengths and limitations

There was clear acknowledgment of the DRI among the GP clinicians in this study, supporting its recognition as a concept. The vignettes and think-aloud method enabled the intended uncertainty to be created and evidenced. It prompted clinicians to articulate their position both before and after the DRI, enabling exploration of any changes. Once experienced, clinicians recognised the concept of DRI and the dilemmas it imposed on their clinical decision making.

Clinicians acknowledged the parallels between DRI in the form of diagnostic tests and clinical scores. However, outside of this study, tests and scores may be perceived differently by clinicians, even though both influence diagnostic probability in the same Bayesian manner.

Despite advertising more widely, the clinicians were drawn from a limited geographical area, and findings could be influenced by local medical cultural norms. Interviews were performed by MS Teams, and not face to face, which could result in the loss of some context. Double-coding was only performed on a subsection of the transcripts, creating the possibility of an interpretation bias. A proportion of clinicians knew the interviewer before recruitment, which could bias towards a particular mindset. Vignette studies raise questions about external validity. There is a concern about a lack of realism and a potential Hawthorne effect,^35^ with a response bias towards idealised or theoretically correct answers. However, studies show that clinicians tend to act similarly in both vignettes and real-world decision making.^36^ A study using consultations with actors would provide a more realistic simulated environment to study DRI.

### Comparison with existing literature

Theme 1 explores the dominant role DRI plays in a consultation. It openly and explicitly presents a differential diagnosis that might not have been discussed with the patient otherwise. This raises the question of how likely (and/or how serious) a differential diagnosis needs to be before it should be discussed with the patient.^37^ There is likely no definitive answer to this question. The timing of discussing specific risks and uncertainties will depend on the clinician’s available time and attitude towards risk, the patient’s anxiety levels and willingness to receive such information, and the actual risk involved. An important discovery was the discomfort clinicians experienced when practising defensive medicine and feeling compelled to do so by the DRI. This is an example of cognitive dissonance,^38^ as the defensive actions conflict with not only a clinician’s reasoning, but also their desire not to worry the patient unnecessarily, and to put the patients’ welfare first. While the relationship between medico-legal fears, defensive medicine, shame, and complaints has been previously described,^39–41^ the DRI may exacerbate the situation by introducing a differential and risk information, that the clinician had no agency over.

Theme 2 explores several dimensions of trust. There is a body of evidence on this topic,^42,43^ but the primary focus is a patient’s trust in clinicians. Mistrust of clinicians is a driver of overtesting and overmedicalisation,^44^ and trust has been described as essential in the management of uncertainty.^45^ What has been less studied is a clinician’s trust in a patient,^44,46^ and this study suggests this might also be crucial. DRI, introduced without clinician discretion, compels a clinician to discuss a particular diagnosis. It not only removes a moment of agency from the clinician but also eliminates a moment of trust between the patient and clinician: that of trusting a clinician to decide if further tests or risk scores are needed.

Theme 3 highlights the contrast between the complex nature of clinical diagnosis and the superficial, yet misleading, certainty provided by test results or clinical scoring systems. This has been described previously.^47,48^ Clinicians often use categorical phrases, such as 'rule out' and 'exclude' when referring to tests and scores, contributing to a societal misconception that medical diagnosis is simple.^49^ It may be that even probabilistic point estimates are inappropriate, and a range of probabilistic values is more appropriate, making explicit the inherent uncertainty within diagnosis.^50,51^

### Implications for research and practice

For those designing or implementing clinical warning systems or pathways that involve routine scores or testing, this study might provide insight into the cognitive challenges that can arise. For practising clinicians, DRI may not be a novel experience, but they may not have recognised its impact on how they feel, think, and make decisions with patients. The increasing fragmentation of primary care,^52,53^ a lack of emphasis on continuity of care,^54^ and the growing trends of private health testing^15,55^ and personal monitoring equipment^56^ are making DRI more prevalent. Tomorrow’s GPs need to prepare to consult in an environment where patients have an impression, be it clinically reasonable or not, from sources such as the internet or AI.^57^ More research is needed into how to incorporate this information into clinical reasoning.

In conclusion, while sometimes offering clinicians a valuable tool to support diagnostic decision making, DRI has the potential to dominate the consultation and cause cognitive dissonance. The findings of this study indicate that clinicians acknowledge the complexity of diagnosis and should be supported to appreciate their own clinical judgement while presenting any risk information related to diagnosis in a nuanced, non-binary manner.

## Data

The dataset relied on in this article is available from the corresponding author on reasonable request.
